# Dispersal Ability Reduces Thermal Specialization and Prevents Climate‐Driven Extinctions in a Neotropical Rainforest

**DOI:** 10.1111/gcb.70399

**Published:** 2025-08-05

**Authors:** Cleber J. N. Chaves, Ana C. Carnaval, Bárbara S. S. Leal, Jessie P. Santos, Erison C. S. Monteiro, Clarisse Palma‐Silva

**Affiliations:** ^1^ Departamento de Biologia Vegetal, Instituto de Biologia Universidade Estadual de Campinas Campinas Brazil; ^2^ City College of New York Biology Department New York New York USA; ^3^ Biology Ph.D. Program The Graduate Center of the City University of New York New York New York USA; ^4^ Instituto Tecnológico Vale Belém Brazil; ^5^ Departamento de Biologia Animal, Instituto de Biologia Universidade Estadual de Campinas Campinas Brazil; ^6^ Instituto Nacional de Pesquisas Espaciais São José dos Campos Brazil

**Keywords:** climate change, climate tracking, distribution range, mobility, thermal tolerance

## Abstract

Dispersal ability is a key factor in determining a species' realized niche. However, it remains unclear whether dispersal ability directly, indirectly, or neutrally affects environmental specialization and species' tolerance ranges. Here, we investigate whether, and how, dispersal ability shapes both the realized and fundamental niches. Focusing on plants, invertebrates, and vertebrates in the topographically complex Atlantic Rainforest—one of the world's top biodiversity hotspots—we also assess how dispersal ability correlates with species' range shifts in response to climate change. Our findings indicate that high‐dispersal species exhibit broader thermal tolerances compared to low‐dispersal taxa, which are often restricted to higher elevations. Projected across geographic space, these data forecast a concerning scenario for species with limited dispersal abilities—particularly low‐dispersal ectotherms—which are expected to face the highest risks of local extinction, even under the milder climate projections for the end of the 21st century. In contrast, species with broader thermal tolerances and higher dispersal capacities are expected to undergo reduced range shifts in response to climate change, particularly under the milder climate projection. Therefore, while the milder projections already indicate high extinction rates in the highlands, the warmest future scenario exacerbates this trend by predicting a substantial influx of high‐dispersal species moving upslope (and southward) that are also expected to be locally affected by climate change. These upward movements are expected to negatively affect native communities closely tied to the forest's mountaintop ecosystems. Given the rapid habitat conversion affecting this and similar landscapes globally, we emphasize the importance of prioritizing low‐dispersal species in biodiversity management. Our results highlight the critical role of dispersal ability in species' resilience to ongoing climate warming, especially in biodiversity‐rich but threatened regions like the Atlantic Rainforest.

## Graphical abstract

We found that species with higher dispersal ability are less specialized in their temperature tolerances and more likely to persist under climate change. By analyzing more than 6 thousand species of plants, invertebrates, and vertebrates distributed in the Atlantic Rainforest, we show that more mobile species can avoid extinction by shifting locations, while less mobile ones evolve narrower thermal niches that increase their vulnerability.

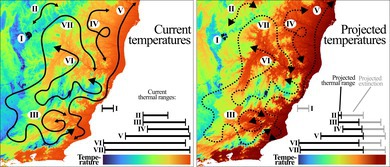

## RESUMO

A habilidade de dispersão é um fator chave na determinação do nicho realizado de uma espécie. No entanto, ainda não está claro se essa habilidade afeta a especialização ambiental e os intervalos de tolerância das espécies de forma direta, indireta ou neutra. Neste estudo, investigamos se, e como, a capacidade de dispersão molda tanto os nichos realizados quanto os fundamentais. Focando em plantas, invertebrados e vertebrados da topograficamente complexa Mata Atlântica—um dos principais hotspots de biodiversidade do mundo—também avaliamos como a habilidade de dispersão se correlaciona com os deslocamentos de distribuição das espécies em resposta às mudanças climáticas. Nossos resultados indicam que espécies com alta capacidade de dispersão apresentam tolerâncias térmicas mais amplas em comparação com táxons de baixa dispersão, frequentemente restritos a altitudes mais elevadas. Quando projetados no espaço geográfico, esses dados preveem um cenário preocupante para espécies com capacidades limitadas de dispersão—particularmente ectotérmicos de baixa dispersão—que devem enfrentar os maiores riscos de extinção local, mesmo sob os cenários climáticos mais amenos projetados para o final do século XXI. Em contraste, espécies com tolerâncias térmicas mais amplas e maiores capacidades de dispersão tendem a sofrer deslocamentos de distribuição mais limitados em resposta às mudanças climáticas, especialmente sob projeções mais amenas. Assim, embora os cenários mais brandos já indiquem altas taxas de extinção nas regiões montanhosas, o cenário futuro mais quente agrava essa tendência ao prever um influxo substancial de espécies altamente dispersoras se deslocando para altitudes mais elevadas (e em direção ao sul), as quais também deverão ser afetadas localmente pelas mudanças climáticas. Esses deslocamentos ascendentes devem impactar negativamente as comunidades nativas associadas aos ecossistemas de topo de montanha da floresta. Diante da rápida conversão de habitat que afeta esta e outras paisagens semelhantes globalmente, enfatizamos a importância de priorizar espécies de baixa dispersão na gestão da biodiversidade. Nossos resultados destacam o papel crítico da capacidade de dispersão na resiliência das espécies ao aquecimento climático em curso, especialmente em regiões ricas em biodiversidade, mas ameaçadas, como a Mata Atlântica.

## 1 Introduction

Species can expand their range only if they overcome environmental barriers and locate suitable conditions for establishment. The importance of dispersal ability—considering both an organism's movement capacity and the spread of its propagules—as a driver of species distribution was first highlighted by the theory of island biogeography (MacArthur and Wilson 1967) and has since been integrated into numerous biogeographical frameworks (Bermingham et al. 1992; Cumming et al. 2012). Together with evolutionary history and species interactions, dispersal ability is pivotal in determining a species' potential to occupy regions that offer the environmental conditions needed for its survival and growth (i.e., the fundamental niche; Soberón and Peterson 2005; Nathan et al. 2008; Cumming et al. 2012). Generally, species with limited dispersal capacities are expected to have smaller realized niches than those with high dispersal abilities, as they access fewer regions within their fundamental niche (e.g., Tovar et al. 2023). However, the extent to which dispersal ability shapes the limits of the fundamental niche remains uncertain (but see McCauley et al. 2014).

**FIGURE 1:**
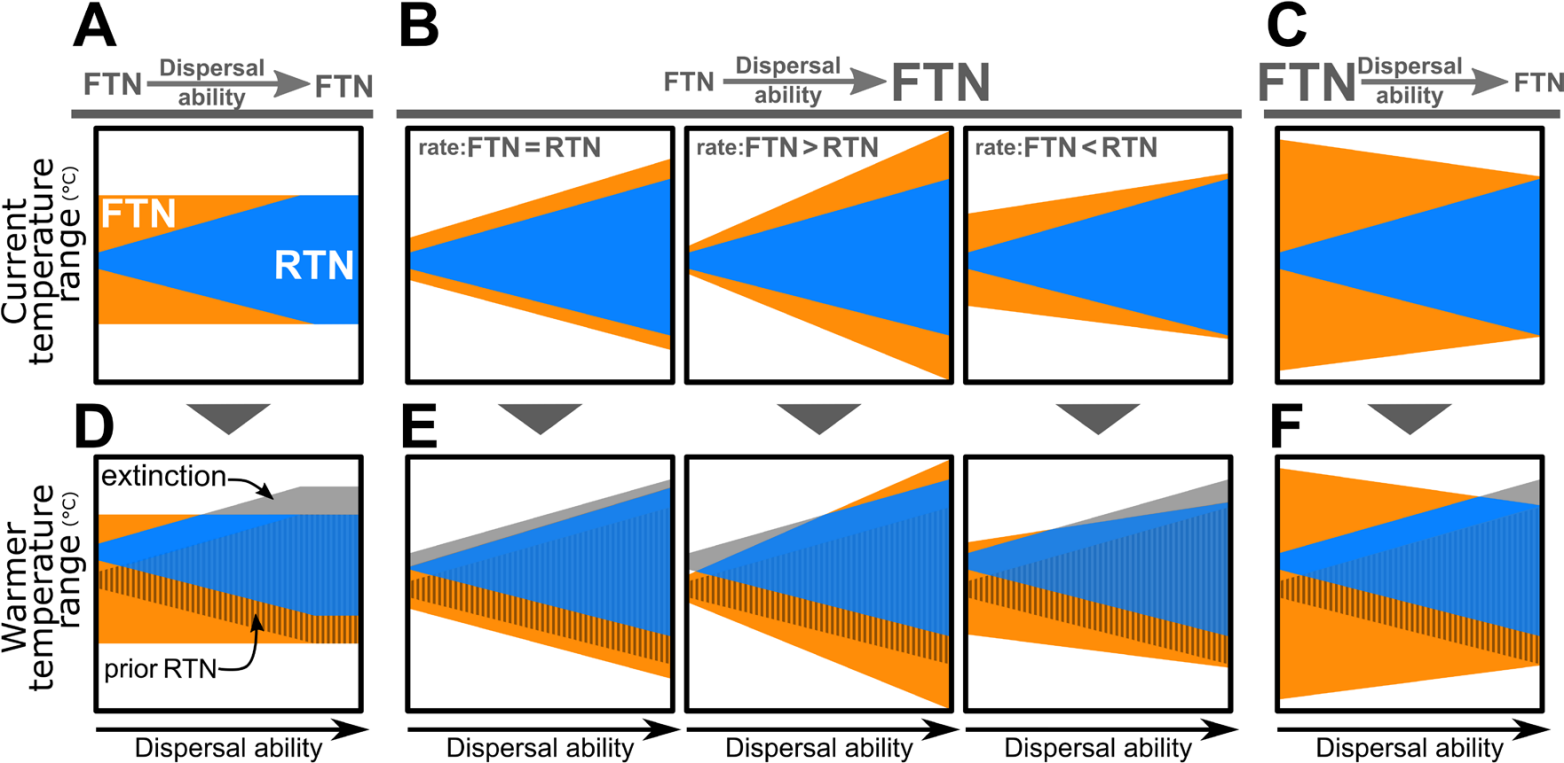
Hypothetical effects of dispersal ability on the breadth of realized thermal niches (RTN, in blue) and fundamental thermal niches (FTN, in orange). The RTN represents the range of temperatures a species occupies, while the FTN represents the range of temperatures a species can physiologically tolerate. We here consider that RTN limits expand with dispersal ability. The graphs illustrate various scenarios in which dispersal ability also influence FTN breadth (indicated in the graph headers by the font size of “FTN” before and after the “dispersal ability” arrows). Scenarios depicted include no effect (A and D), positive effects (B and E), and negative effects (C and F). Among the positive rates (i.e., columns B and E), the influence of dispersal ability may expand the FTN at a similar rate (rate: FTN = RTN), a greater rate (rate: FTN > RTN), or a lesser rate (rate: FTN < RTN) relative to the RTN expansion. Dispersal ability may affect a species' thermal niche breadth by enabling access to diverse environments and exposure to varied temperature regimes. D‐F indicate expected climate change impacts: As temperatures rise in a species' prior range (hatched areas), local extinctions (gray) may occur depending on its FTN. In such cases, high dispersal ability could help species track cooler areas, while low dispersal capacity may leave species vulnerable to climate change without access to suitable conditions.

Dispersal ability is thought to influence tolerance limits by shaping selective pressures on organisms (Huey et al. 2002; Angilletta Jr et al. 2006; Muñoz et al. 2019; Figure 1). Low dispersal ability may lead to population isolation and reduced gene flow, which can foster local specialization and result in a narrower fundamental niche (Lester et al. 2007; Lavery et al. 2020). Conversely, high dispersal ability, by facilitating access to a broader range of environments, is associated with wider physiological limits (i.e., broader fundamental niches) and more generalist strategies (Huey et al. 2002; Claramunt et al. 2012; Faurby and Antonelli 2018).

The dispersal ability of organisms would be related to increased tolerance limits and realized niches in different magnitudes (Figure 1B). But an alternative perspective suggests that dispersal ability enables species to buffer against environmentally imposed selection on physiological tolerance, thus preventing the expansion of their fundamental niche (Huey et al. 2002; Angilletta Jr et al. 2006; Gunderson and Stillman 2015; Muñoz and Bodensteiner 2019; see Figure 1C). In this view, high dispersal ability compensates for narrow tolerance limits by allowing species to avoid the negative effects of environmental changes, while wide tolerance limits can mitigate the challenges posed by low dispersal ability (Figure 1C). This interplay between dispersal ability and thermal tolerance has been demonstrated in insects (e.g., Bowler and Terblanche 2008; Uyi et al. 2017), but its effectiveness in explaining broad geographical patterns of biodiversity across diverse taxonomic groups remains untested. Additionally, the downstream impacts of this relationship on predicting species' responses to future climates are largely unknown.

Regardless of whether dispersal ability increases or decreases tolerance limits, it is expected to directly influence how species respond to climate change (Figure 1) and plays a critical role in reshaping biodiversity patterns in a warmer world. This influence is particularly concerning in tropical megadiverse regions with low seasonality, which host a large number of thermal specialist species (Janzen 1967; Polato et al. 2018; Cuesta et al. 2019; Rahbek et al. 2019). The situation is even more alarming in tropical areas like the Atlantic Rainforest, where climate change exacerbates the effects of decades of intense human pressure that have reduced South America's second‐largest rainforest—one of the world's top five biodiversity hotspots (Myers et al. 2000)—to numerous small and isolated fragments (Ribeiro et al. 2009; Joly et al. 2014).

In this study, we investigate how dispersal ability correlates with the limits of realized and fundamental niches among various species in the Atlantic Rainforest, exploring how these factors may shape biological responses to future climate change (here, focusing in temperature shifts). Our main hypotheses propose that increasing dispersal ability is associated with broader fundamental niches, leading to larger realized niches and, consequently, lower vulnerability to climate change (H1; Figure 1B,E). Alternatively, under a climate change scenario, a compensatory mechanism may exist between dispersal ability and fundamental niche size, where highly dispersive species can access habitats with optimal conditions, while low‐dispersive species develop broader environmental tolerances due to their limited ability to escape unfavorable conditions (H2; Figure 1C,F). To test these hypotheses, we analyzed realized and fundamental thermal niches (hereafter defined as RTN and FTN, respectively) of multiple species from the Atlantic Rainforest by compiling data on the spatial ranges and thermal tolerances of birds, terrestrial and flying mammals, amphibians, snakes, ants, butterflies, trees, and epiphytes. To facilitate the comprehension of general patterns, these organisms were categorized into four distinct groups: endothermic vertebrates (mammals and birds), ectothermic vertebrates (amphibians and snakes), arthropods (ants and butterflies), and plants (trees and epiphytes). In the absence of extensive ecological data and comparable traits among these groups, we used species range size as a proxy for dispersal ability, as a positive and causal relationship between these two variables has been demonstrated in various organisms (McCauley et al. 2014; Alzate and Onstein 2022; Arroyo‐Rodríguez et al. 2023). By leveraging our understanding of dispersal ability and RTN and FTN limits of the local biota, we then project the potential impacts of climate change on the biodiversity patterns of species in the Atlantic Rainforest.

## 2 Materials and Methods

### 2.1 Latitude and Elevation Effects on Thermal Range Limits

To characterize the realized thermal niche (RTN), we compiled curated occurrence data for organisms distributed in the Atlantic Forest. The final dataset consists of published information on community composition and distribution for various plants and animals, along with floristic inventory data from the NeoTropTree database (see Table 1 for references). Out of a total of 600,143 occurrence records for birds, mammals, amphibians, snakes, ants, butterflies, trees, and epiphytes, we retained 560,965 records from 6732 species that had at least five occurrences with precise coordinates (Table 1). To assess the temperature extremes reported across their ranges, we extracted the maximum temperature of the warmest month (Bio 5) and the minimum temperature of the coldest month (Bio 6) from the CHELSA database (Karger et al. 2020) for all retained species occurrence records. We defined the warm and cold limits of each species' RTN as the highest and lowest temperature values obtained from the Bio 5 and Bio 6 bioclimatic rasters, respectively. To investigate how strong RTN limits are related to environmental gradients influenced by latitude and elevation limits in species' ranges, we created linear models with the warm and cold ends of species' RTN as dependent variables. We included the interaction between the lowest and highest latitude and elevation of the species' ranges as independent variables. Finally, we employed ANOVA to test the significance of the models.

**TABLE 1:** Total and retained numbers of records for each group, compiled from the literature, are provided to estimate species range sizes. Only species with at least five precise records were retained for this analysis.

Organism	Group	Total records	Retained records	Retained species	References
Birds	Endotherms	33,391	31,919	549	Rodrigues et al. (2019)
Bats	Endotherms	942	852	38	Muylaert et al. (2017)
Primates	Endotherms	8121	2079	25	Culot et al. (2019)
Mid‐large Mammals	Endotherms	1772	1687	62	Souza et al. (2019)
Small Mammals	Endotherms	2620	1652	63	Bovendorp et al. (2017)
Snakes	Ectotherms	72,440	71,706	217	Nogueira et al. (2019)
Amphibians	Ectotherms	17,619	9985	332	Vancine et al. (2018)
Butterflies	Arthropods	6254	6069	203	Santos et al. (2018)
Ants	Arthropods	52,343	51,512	773	Silva et al. (2022)
Trees	Plants	315,372	313,248	3254	Oliveira‐Filho (2017)
Epiphytes	Plants	89,269	70,256	1216	Ramos et al. (2019)
Total	—	600,143	560,965	6732	—

### 2.2 Species Range Size and Fundamental Thermal Niche Estimation

We estimated species‐specific range sizes by calculating the area of the polygon encompassing all species records (i.e., the convex hull) using the ‘geosphere’ R package (Hijmans et al. 2017). To understand how records are aggregated across species' ranges, we calculated the Clark and Evans Aggregation Index with the Cumulative Distribution Function method (CDF), using the ‘spatstat’ R package (Baddeley et al. 2015). This index calculates the ratio between the observed mean nearest‐neighbor distance and the expected distance under a random distribution (Clark and Evans 1954); values less than 1 indicate clustering, values around 1 indicate randomness, and values greater than 1 indicate regular or dispersed patterns. To characterize the species' fundamental thermal niche (FTN), we compiled experimental studies reporting heat or cold tolerance for species in the Atlantic Rainforest. We began by searching empirical data for the 6732 species using the *GlobTherm* database (Bennett et al. 2018). We found 26 papers with the thermal tolerance data of 58 species (36 and 51 species with heat and cold tolerance, respectively). Additionally, we conducted a systematic literature survey on the *Web of Science* (webofknowledge.com) for indexed articles published from 1900 onward, using the following keyword string:

‘ALL = (“thermal tolerance” OR thermotolerance OR “cold tolerance” OR “heat tolerance” OR “frost tolerance” OR “frost damage” OR “heat damage” OR “cold damage” OR “heat resistance” OR “frost resistance” OR “cold resistance”) AND ALL = (butterfly* OR mammal* OR bat* OR primate* OR monkey* OR bird* OR plant* OR tree* OR epiphyte* OR amphibian* OR frog* OR ant* OR snake* OR viper* OR serpent*) AND ALL = (“South America” OR “Central America” OR Brazil OR Argentina OR Uruguay OR Paraguay OR Bolivia OR Chile OR Peru OR Colombia OR Venezuela)’.

We reviewed 799 papers from this survey and excluded those that did not provide experimental data on thermal tolerance for the species of interest. From the 19 remaining studies, we extracted the upper and/or lower values of critical or lethal temperatures for 129 species (117 taxa provided upper tolerance temperatures, and 38 taxa provided lower tolerance temperatures). In total, combining data from the GlobTherm database and the subsequent systematic literature survey, we obtained thermal tolerance data for 187 species (153 with upper tolerance temperatures and 89 with lower tolerance temperatures; see Table S1).

Author‐provided metrics characterizing species tolerance varied across studies, reflecting differences in species physiology and the methodologies employed. Consequently, we compiled a dataset detailing species' thermal tolerance based on several metrics: lethal temperatures (i.e., temperature leading to 50% mortality, or LT_50_), critical temperatures under regular locomotion (boundary temperatures, CT), chlorophyll fluorescence (T_C_ and T_50_), and the endothermic thermal neutral zone (TNZ). For studies that did not report a specific metric to describe the species' thermal tolerance but provided raw data as Supporting Information or in graphical form, we calculated thermal limits using T_50_ for plants (Valladares and Pearcy 1997) and CT_max_ for ectotherms (Hutchison 1961). Across the literature survey, if a study reported multiple thermal tolerance values under non‐stressful conditions (e.g., during drought stress), we used the average value for the species.

### 2.3 Effect of Dispersal Ability on Species' Niches

To test how dispersal ability impacts the species' RTN and FTN, we constructed two distinct linear models using the warm and cold ends of each species' RTN or FTN as dependent variables, with species range size (used as a proxy for dispersal ability) as a predictor. We assessed model significance using ANOVA. To evaluate whether dispersal ability has a similar, greater, or lesser impact on FTN compared to RTN, we employed the ‘*lsmeans*’ package (Lenth 2016) to statistically compare the slopes of FTN and RTN based on dispersal ability.

### 2.4 Species Vulnerability to Climate Change and the Effects on the Atlantic Rainforest Biodiversity

To assess the vulnerability of each species to future climate change and determine whether environmental shifts will differentially affect species with varying dispersal abilities, we characterized the expected thermal changes in each species' range based on their current FTN and RTN limits. For each occurrence record, we compiled the maximum expected temperatures (Bio 5) and minimum expected temperatures (Bio 6) projected by the average of five global circulation models available in the CHELSA_[CMIP5] database (CMCC‐CM, MIROC5, Access1‐0, MPI‐ESM‐MR, and CESM1‐BGC; Karger et al. 2020). These projections consider a moderate (RCP 4.5) and the warmest climate scenario for the end of the 21st century (i.e., RCP 8.5), which represents an average increase of 3°C in the maximum temperature of the Atlantic Rainforest by 2061–2080. To test whether the magnitude of the expected thermal changes within the current species range is related to dispersal ability, we constructed linear models using range sizes as predictors and assessed their significance using ANOVA.

To assess the impact of projected climate change on biodiversity patterns in the Atlantic Rainforest, we analyzed local extinctions and potential migratory routes for the 153 species with available heat tolerance data, focusing on projected temperatures (RCP 8.5 and RCP 4.5) within the Atlantic Rainforest's boundaries. Local extinction was defined as occurring when the projected maximum temperature at an occurrence site exceeds the species' current thermal tolerance limits. We identified potential migratory routes by determining the nearest coordinates (target points) from each local extinction coordinate (source points) with thermal conditions within the species' tolerance limits, using the ‘RANN’ package (Jefferis 2024).

To evaluate how these factors influence overall biodiversity patterns, we grouped current and projected occurrence coordinates (considering migratory routes) to estimate species richness per spatial unit in a coarser scale raster (~30 km). We then interpolated current and projected species richness based on these thermal tolerance limits using thin‐plate spline regression (Hutchinson and Gessler 1994) via the ‘fields’ package (Nychka et al. 2015) and the ‘raster’ package (Hijmans 2025). In these analyses, we incorporated current and projected RCP 8.5 and RCP 4.5 values of bioclimatic variables Bio 5 and Bio 6 as covariates within the Atlantic Rainforest region.

To examine whether projected temperatures will affect species differently based on dispersal abilities, we built linear models with two dependent variables: the proportion of local extinctions and the optimal migratory route distance (i.e., the shortest path between source and estimated target points, calculated using the ‘raster’ package; Hijmans 2025). To assess if biodiversity pattern changes vary by dispersal ability, we grouped species into five categories with equal species counts based on range sizes, using the ‘*Hmisc*’ package (Harrell and Frank 2019). The groups were defined by range size intervals: (1) 33 to 1.8 × 10^5^ km^2^; (2) 1.8 × 10^5^ to 3.5 × 10^5^ km^2^; (3) 3.5 × 10^5^ to 6.4 × 10^5^ km^2^; (4) 6.4 × 10^5^ to 1.0 × 10^6^ km^2^; (5) 1.0 × 10^6^ to 2.1 × 10^6^ km^2^. We then applied the interpolation method described previously for each group to analyze biodiversity pattern shifts across these dispersal categories.

## 3 Results

### 3.1 Latitude and Elevation Effects on Thermal Range Limits; and Species Range Estimation

**FIGURE 2:**
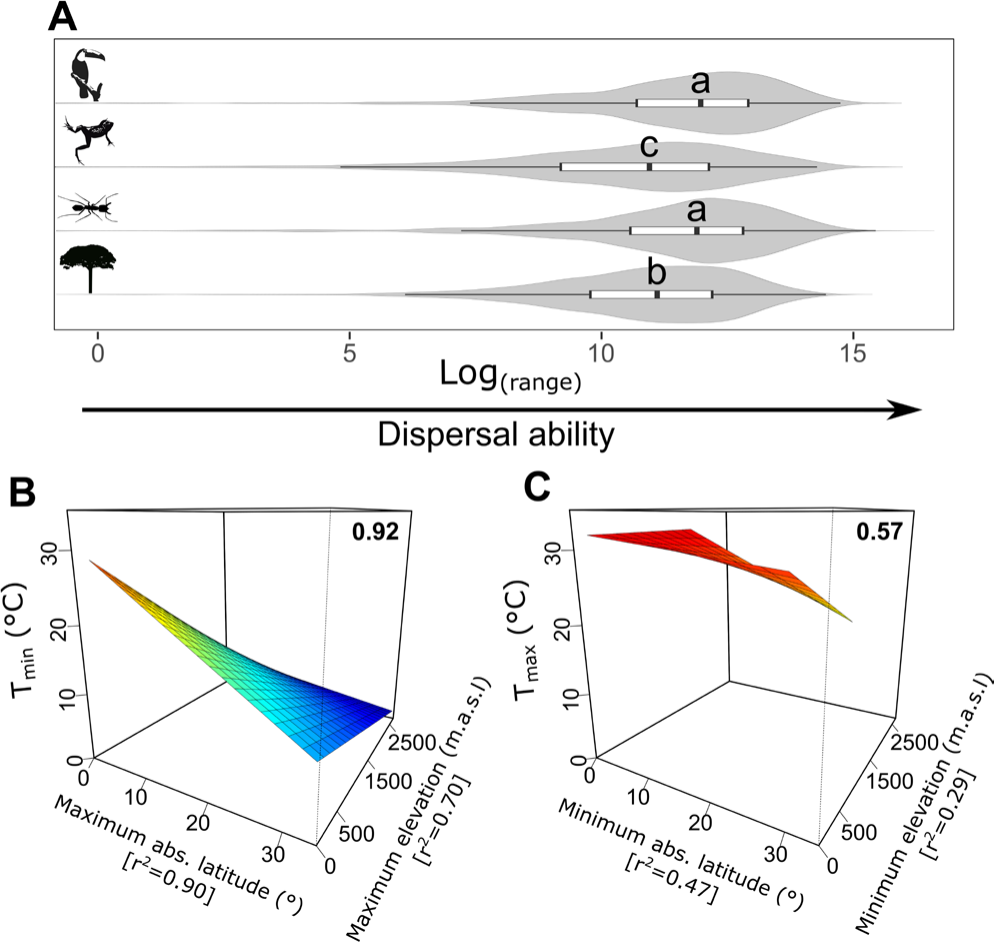
Effects of latitude and elevation on species‐specific thermal limits of realized thermal niches (RTN). Panel A shows the distribution of species range sizes by organism groups analyzed (endotherms, ectotherms, arthropods, and plants, from top to bottom), with similar letters next to violin plots indicating statistically similar means (*p* > 0.05). Panels B and C present best‐fit linear models, with bold numbers indicating the models' *R* ^2^ values, illustrating the relationships between extreme latitudes and elevations occupied by each species and their minimum (*T*_min_; B) and maximum realized thermal limits (*T*_max_; C). Warmer colors represent higher expected *T*_min_ (B) and *T*_max_ (C) values. The data consist of 560,965 occurrence records across 6732 species and temperature estimates from the CHELSA database (Karger et al. 2020).

Across all species, range size varied widely, from approximately 0.03 km^2^ for *Hatiora cylindrica* , an epiphytic cactus, to around 2.5 × 10^6^ km^2^ for *Vireo chivi*, a bird. Most species had highly clustered occurrence records, with most displaying aggregation indices below 0.3 (Figure S3). Endotherms and arthropods exhibited significantly broader ranges compared to ectotherms (Figure 2A). Temperature breadth, or the variation in species' thermal limits, correlates with the latitudinal and altitudinal variation within the ranges of Atlantic Rainforest species (Figure 2). The minimum air temperature within species ranges (*T*_min_) tends to occur at higher elevations or latitudes, with a strong relationship to spatial variables (*R* ^2^ = 0.924, *F* = 1682, *p* < 0.001; Figure 2B). Maximum air temperature within ranges (*T*_max_), however, showed a weaker relationship with latitude and elevation (*R* ^2^ = 0.566, *F* = 181.1, *p* < 0.001; Figure 2C).

### 3.2 Effect of Dispersal Ability on Species' Realized and Fundamental Niches

**FIGURE 3:**
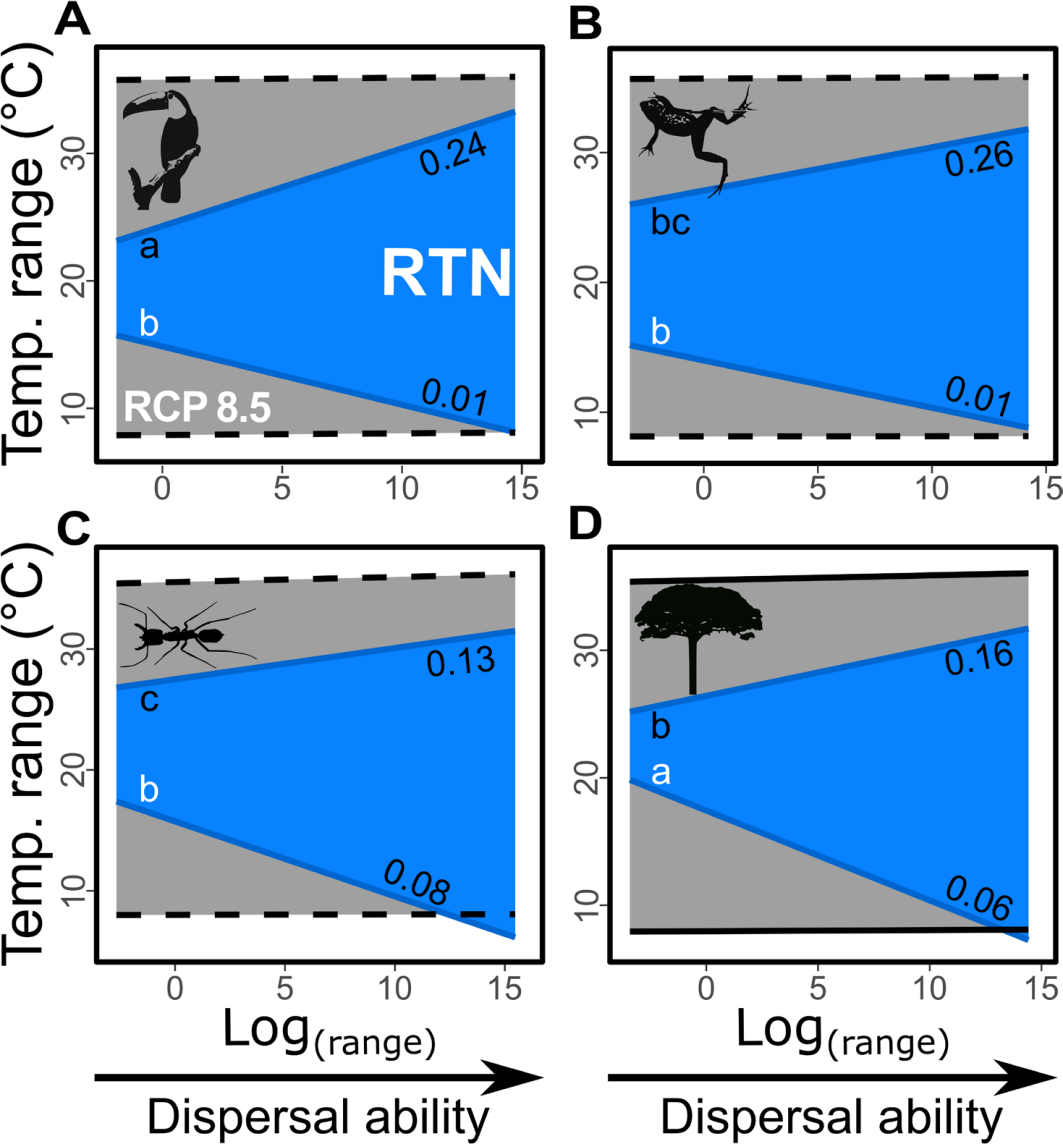
The effect of dispersal ability on the realized thermal niche and projected climate‐driven thermal shifts. Relationships between dispersal ability (measured as range size) and the realized thermal niche (RTN) limits (blue) are shown for approximately 6700 Atlantic Rainforest species, categorized as endotherms (A), ectotherms (B), arthropods (C), and plants (D). Additionally, expected thermal shifts under the most severe climate change scenario (RCP 8.5; Karger et al. 2020) are represented in gray. Continuous and dashed lines denote significant (*p* < 0.05) and non‐significant (*p* > 0.05) relationships with the RTN limits (blue lines) and RCP 8.5 projections (gray lines), respectively. *R* ^2^ values at the right end of each blue line show the strength of the relationship between range size and thermal limits. Similar letters indicate statistically similar slopes between regressions, with black letters for warm limits and white letters for cold limits.

Species with greater dispersal abilities, indicated by larger range sizes, tend to have broader realized thermal niches (RTN), showing significant positive relationships between dispersal ability and both the warm and cold limits of species' RTNs (*p* < 0.05; see blue curves in Figure 3). Among all organism groups, the relationship is notably stronger for warm limits than for cold limits, with the highest regression slope between dispersal ability and warm RTN limits found in endotherms (*p* < 0.05; Figure 3A). For cold RTN limits, plants exhibit the steepest slope (*p* < 0.05; Figure 3D).

**FIGURE 4:**
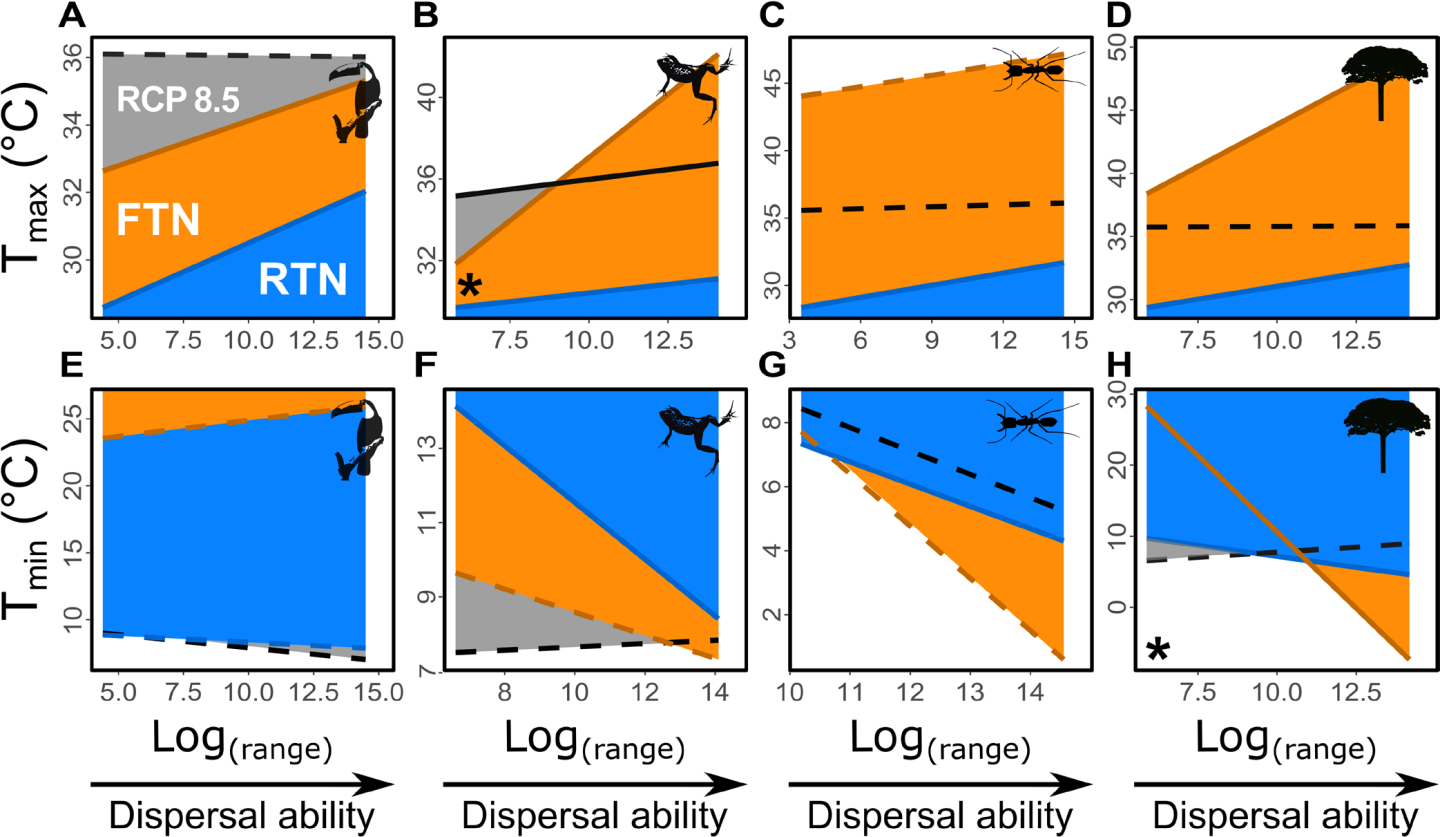
The effect of dispersal ability on realized (RTN, in blue) and fundamental thermal niches (FTN, in orange). Relationships between dispersal ability (measured as range size) and the warm (A–D) and cold (E–H) thermal limits of the RTN and FTN for 180 species from the Atlantic Rainforest, including endotherms (A, E), ectothermic vertebrates (B, F), arthropods (C, G), and plants (D, H). Gray lines and shaded areas represent projected shifts in RTN limits under the most severe climate scenario (RCP 8.5; Karger et al. 2020). Solid and dashed lines signify significant (*p* < 0.05) and non‐significant (*p* > 0.05) correlations between niche limits and species' range size. Asterisks denote significant differences (*p* < 0.05) between RTN and FTN slopes.

Similarly, higher dispersal ability is associated with broader fundamental thermal niches (FTN), where FTN temperature ranges increase as dispersal ability grows (Figure 4). FTN ranges are generally broader than RTN for most species with thermal tolerance data. However, significant differences (*p* < 0.05) between FTN and RTN regression slopes—when compared against dispersal ability—were only evident for the warm limits of ectotherms and the cold limits of plants (Figure 4B,H). Notably, cold RTN limits were lower than FTN limits across all endotherms, as well as for small‐range arthropods and plants (Figure 4E,G,H).

### 3.3 Species Vulnerability to Climate Change

**FIGURE 5:**
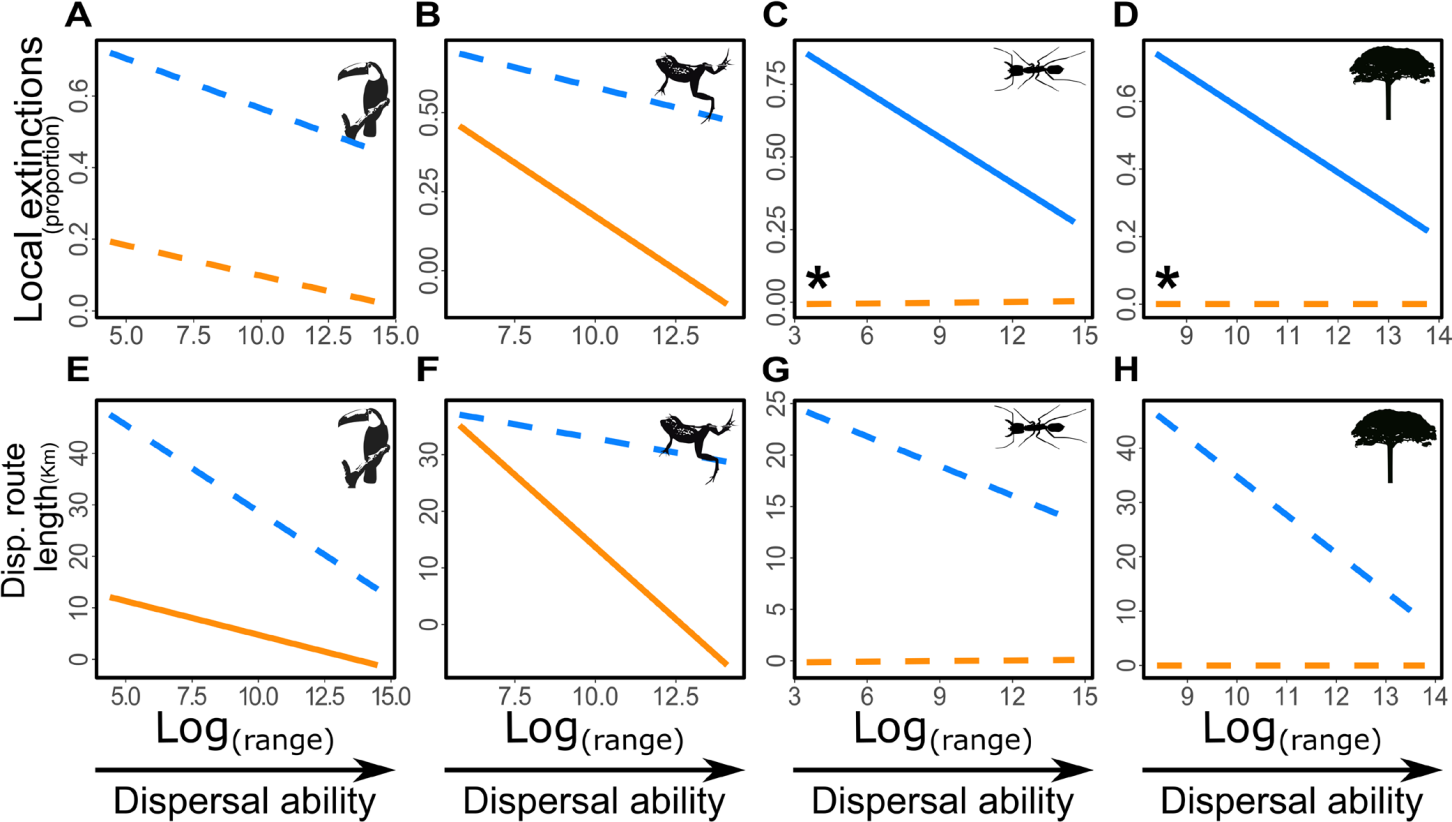
Relationships of dispersal ability with projected local extinction rates and dispersal route lengths. Dispersal ability (range size) is related to estimated local extinctions (A‐D) and projected dispersal route lengths (E–H) in 180 species of Atlantic Rainforest endotherms (A and E), ectothermic vertebrates (B and F), arthropods (C and G), and plants (D and H). Calculations consider the impact of the most extreme climate change scenario (RCP 8.5; Karger et al. 2020) on species occurrences, within the current limits of realized (RTN, blue lines) and fundamental thermal niches (FTN, orange lines). Solid lines indicate significant relationships (*p* < 0.05), while dashed lines represent non‐significant relationships (*p* > 0.05). Asterisks denote significant differences (*p* < 0.05) between RTN and FTN projections.

Under projected climate conditions (RCP 8.5), temperatures will surpass the warmest limits currently observed within the ranges of Atlantic Rainforest species (Figure 3), with some species also likely to experience temperatures below their present‐day cold limits. The magnitude of these temperature shifts is independent of dispersal ability (i.e., range size; see Figures 3 and 4). However, low‐dispersal species (with more restricted ranges) are expected to face greater impacts from climate change (Figure 5). Specifically, a negative relationship exists between dispersal ability and both the optimal migration route length to reach suitable habitats and the proportion of local extinctions, although this effect is weaker in ectotherms compared to other groups (*p* < 0.05; Figure 5E–H).

### 3.4 Climate Change Effects on Atlantic Rainforest Biodiversity

**FIGURE 6:**
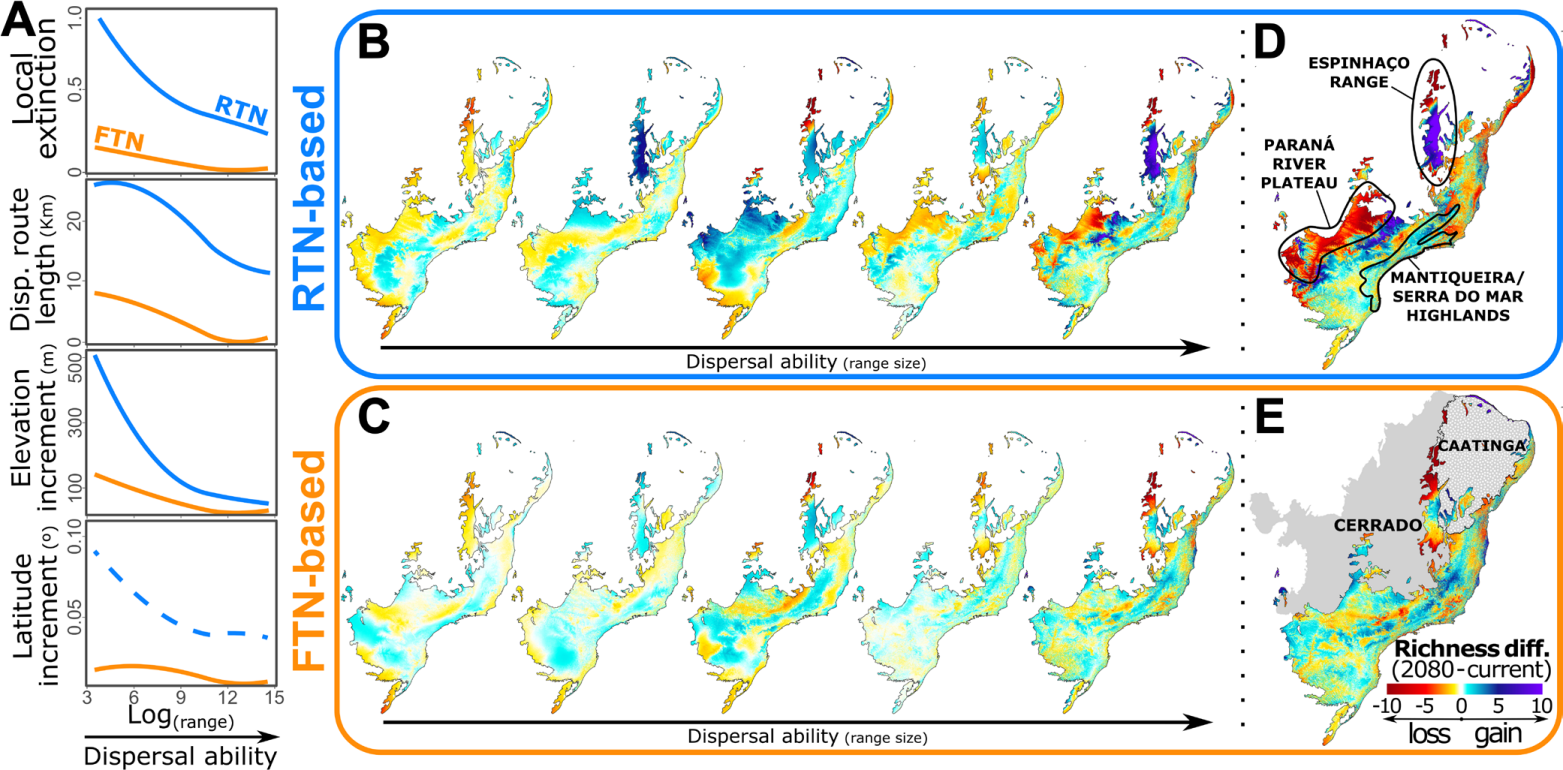
The effects of dispersal ability on biodiversity shifts in the Atlantic Rainforest due to climate change (RCP 8.5). Chart A presents predicted local extinction rates and climate tracking metrics—specifically dispersal route lengths, elevation and latitude increments—relative to dispersal ability (i.e., range size). The continuous and dashed curves denote significant (*p* < 0.05) and non‐significant polynomial relationships for the realized (RTN; in blue) and fundamental thermal niches (FTN; in orange) of various species. Maps B‐E depict the current and projected biogeographic patterns of species richness in the worst climate change scenario for the end of the 21st century (RCP 8.5; Karger et al. 2020). The current species richness maps are based on distribution data for 180 Atlantic Rainforest species with available physiological and occurrence data, while the projected maps reflect shifts arising from climate tracking indicated in Chart A. Maps in B and C differentiate expected species gain (cold colors) and loss (warm colors) by classifying species into five groups based on similar range sizes. Maps D and E present the overall patterns of species richness changes without regard to dispersal ability.

Considering the RTN limits, our results indicate that Atlantic Rainforest species will lose, on average, 13% of their current range under RCP 4.5% and 27% under RCP 8.5. In contrast, the FTN limits project local extinction rates of 8% under RCP 4.5% and 8.5% under RCP 8.5. While both RCP 8.5 and RCP 4.5 future climate projections predict a general shift in species distributions towards higher elevations and more southern latitudes, we found substantial differences between the two scenarios (Figures 6A and S4). For instance, under the RTN limits, the average dispersal distance required to track suitable climate conditions is 4.5 km under RCP 4.5, but increases dramatically to 12 km under RCP 8.5. This is accompanied by an average shift in latitude from 0.02° to 0.05° south and an elevation increase from 21 m to 60 m. Similar trends are observed under the FTN limits, although the differences between RCP 4.5 and RCP 8.5 are less pronounced. In this case, average dispersal distances increase from 0.30 to 2 km, with latitude shifting from 0.002° to 0.015° south and elevation rising from 2.5 to 6.8 m.

Species with low dispersal ability (i.e., those with the smallest range sizes) are projected to experience the highest local extinction rates in the Atlantic Forest under future climatic conditions (RCP 8.5 and 4.5; Figure 6A and S4). Under RCP 8.5, in particular, to track suitable climates based on their RTN limits, these species may need to disperse over distances close to 30 km, ascend approximately 500 m in elevation, or shift around 0.1° southward in latitude, on average (Figure 6A). In contrast, species with high dispersal ability are expected to undergo smaller shifts, requiring only a fraction of the elevation change necessary for low‐dispersal taxa, averaging about one‐tenth of the elevation increment predicted for low‐dispersal species (Figure 6A). A similar, though less pronounced, trend appears when predictions are made using FTN limits (Figure 6A).

Projected range shifts under the RCP 8.5 scenario are expected to significantly alter biodiversity patterns across the Atlantic Forest (Figure 6B–E). RTN‐based predictions indicate that regions, such as the Paraná River Plateau, the Espinhaço Range, and the Mantiqueira/Serra do Mar highlands will undergo substantial biotic changes (Figure 6D). Specifically, the Paraná River Plateau and the northern Espinhaço Range are projected to experience high extinction rates, whereas the highland areas—particularly the southern Espinhaço Range and the Mantiqueira/Serra do Mar complex—are expected to support climate‐tracking species across most dispersal levels (Figure 6B,D). However, species with intermediate dispersal abilities may also be drawn to the Paraná River Plateau, while those with the lowest dispersal capacities, often already confined to highlands, are likely to face higher extinction rates in the Espinhaço Range (Figure 6B). Under the RCP 4.5 scenario, the smaller scale of changes—considering local extinctions and tracking routes (Figure S4A)—suggests a less pronounced impact on biodiversity patterns compared to RCP 8.5 (Figure S4B). Notably, the highlands (particularly, the Paraná River Plateau, the Espinhaço Range, and some areas of the Mantiqueira/Serra do Mar) are less expected to serve as primary destinations for climate‐tracking species under RCP 4.5, thereby increasing the projected species deficit compared to the RCP 8.5 scenario.

The FTN‐based predictions display range shift patterns that differ from those based on RTN, particularly under the RCP 8.5 scenario (Figure 6B–E). While both models agree on high extinction rates in the northern Espinhaço Range, the FTN model also forecasts significant extinction rates in the southern tip of the Espinhaço Range and in forest remnants within the semi‐arid Caatinga biome. Regarding climate‐tracking species, the FTN projections largely align with the RTN model, identifying critical areas such as the highlands in the middle of the Espinhaço Range—which are important for species with varying dispersal abilities, except for those with very low dispersal capacity—and the highlands of the Mantiqueira/Serra do Mar complex, which are expected to be vital for climate‐tracking species regardless of dispersal ability. Under the RCP 4.5 scenario, the main difference between the FTN‐ and RTN‐based projections lies in the overall reduced extent of species deficit in the Espinhaço Range and Paraná River Plateau (Figure S4).

## 4 Discussion

Our results revealed a significant correlation between dispersal ability and both the realized (RTN) and fundamental thermal niches (FTN) of various species, with notable distinctions among different organism groups. We hypothesized that greater dispersal ability would lead to broader thermal niches, thereby enhancing species resilience to climate change. In the following sections, we will delve into how these relationships manifest across taxa, exploring how differences in physiology and behavior influence thermal tolerance and dispersal dynamics. We will also examine the implications for species vulnerability in the context of climate change, highlighting how certain groups may face heightened risks of extinction. Lastly, we will discuss the potential consequences of these shifts on biodiversity patterns within the Atlantic Rainforest ecosystem, emphasizing the importance of understanding these dynamics for effective conservation strategies.

### 4.1 Effect of Dispersal Ability on Species' Realized and Fundamental Niches

We observe a significant relationship between the range of the RTN of Atlantic Forest species and regional variations in latitude and elevation (Figure 2), as well as with dispersal ability (Figure 3). Notably, cold range limits exhibit a strong correlation with variations in latitude and elevation (*R* ^2^ ≈ 0.9), while fluctuations in warm limits are more effectively explained by species' dispersal ability. This finding aligns with previous studies indicating that lower thermal limits of species distributions are closely linked to thermal gradients associated with latitude and elevation (Araújo et al. 2013; Rezende et al. 2014; Perez and Feeley 2020). Furthermore, our results demonstrate that higher dispersal ability (i.e., larger range size) is associated with broader FTNs in the Atlantic Rainforest. This challenges the hypothesis that greater dispersal ability buffers environmentally imposed selection and constrains the fundamental niche, as suggested by some studies (e.g., Huey et al. 2002; Angilletta Jr et al. 2006; Gunderson and Stillman 2015; Muñoz and Bodensteiner 2019). Nonetheless, further research is necessary to determine whether dispersal ability influences thermal tolerance at finer spatial scales than those considered in this study.

Our findings also indicate that physiology or behavior may play a crucial role in mediating the impact of dispersal ability on a species' thermal niche. For example, the absence of thermoregulatory behavior in plants may account for their ability to withstand much lower temperatures compared to animals with similar dispersal abilities (Figures 3D and 4H). Conversely, the high mobility of certain endotherms, such as large mammals and birds, may allow them to temporarily occupy environments with seasonal suboptimal conditions (e.g., Lamb et al. 2020; Parnell et al. 2020), which could explain the steep increase in their warm range limits (Figure 3A) or the colder limits of their ranges relative to their physiological tolerances (Figure 4E). This aligns with previous studies suggesting that endothermy facilitates intermittent exploration of environments with thermal conditions outside a species' thermal tolerance (McNab 2012; Khaliq et al. 2014). However, we cannot discount the possibility that our study may not have fully captured the extent of physiological tolerance to cold conditions, particularly for endothermic organisms, given the metrics and data used in these analyses.

### 4.2 Species Vulnerability to Climate Change

Considering the limits of both the FTN and RTN, our modeling approach indicates that future climates will present a particularly challenging scenario for species with lower dispersal ability in the Atlantic Rainforest. These findings align with other studies suggesting that spatially restricted species are more vulnerable to climate change in tropical regions (e.g., Engler et al. 2009; Cuesta et al. 2019). However, the responses to climate change vary across different organism groups. In the Atlantic Forest, the high tolerance to warm conditions exhibited by arthropods and plants makes them less vulnerable compared to vertebrate ectotherms with similar dispersal abilities (range size; Figure 4C,D). Additionally, our results suggest that the longer dispersal routes required for climate tracking in the Atlantic Rainforest pose further challenges for ectotherms, especially for species with low dispersal abilities. Consequently, the highest extinction rates due to climate change in this ecosystem are anticipated for low dispersal ectotherms, corroborating findings from other studies (e.g., Araújo et al. 2013; Gunderson and Stillman 2015; Polato et al. 2018).

### 4.3 Climate Change Effects on Atlantic Rainforest Biodiversity

Climate‐driven extinctions and climate tracking—quantified here as optimal dispersal routes (Figure 5)—will likely lead to southward and, predominantly, upward shifts in the distribution of Atlantic Rainforest species (Figure 6A). Similar patterns are increasingly documented worldwide (e.g., Kearney et al. 2009; Walther 2010; Bush et al. 2018; Hagedorn et al. 2019). The elevation shifts in distribution patterns, as predicted in this study, are associated with an increased risk of extinction due to competition between resident and climate‐tracking species, as well as reductions in available settlement area at higher elevations (Alexander et al. 2015; Cuesta et al. 2019; Elsen et al. 2020). Given the generally narrower limits of FTN and RTN in low‐dispersal species, which are often constrained topographically, it is not surprising that these species are expected to undergo the most significant changes in biodiversity within the Atlantic Rainforest.

Our RTN‐ and FTN‐based models identify distinct regions of the Atlantic Rainforest as being potentially affected by shifts in species richness. The RTN‐based model highlights the northeastern coast and the Paraná River Plateau as areas facing the highest extinction risks, particularly under the most extreme warming scenario, RCP 8.5 (Figure 6D). Notably, the Paraná River Plateau is among the most disturbed and least protected areas within the Atlantic Rainforest, despite its critical role in connecting moist and semi‐deciduous forests between the Atlantic Forest and the Cerrado biomes (de Albuquerque et al. 2011; Souza et al. 2020). The impacts of climate change predicted by the RTN‐based model, combined with ongoing anthropogenic pressures in the region, underscore a worrying outlook for biodiversity in this transitional zone.

The highlands of the Atlantic Rainforest—especially the Espinhaço Range and the Mantiqueira/Serra do Mar complex—are notably predicted by the RCP 8.5 projections to receive an influx of climate‐tracking species, which may introduce novel competitors into montane communities and potentially lead to catastrophic ecological consequences (Alexander et al. 2015). The Espinhaço Range, one of the oldest mountain systems on Earth, has historically served as an interglacial refugium due to its climatic stability (Alexander et al. 2015; Alkmim and Fo 1998; Pedreira and De Waele 2008; Bonatelli et al. 2014; Barbosa et al. 2015; Silveira et al. 2016; Dantas‐Queiroz et al. 2021, 2023), and, as the second‐largest mountain range in South America after the Andes, it supports exceptionally high levels of plant diversity—around 17% of Brazil's flora (Fernandes et al. 2018)—as a result of evolutionary processes promoting the diversification of narrowly endemic lineages (Colli‐Silva et al. 2019; Vasconcelos et al. 2020; Dantas‐Queiroz et al. 2021, 2023). Similarly, the Mantiqueira/Serra do Mar complex, which includes some of Brazil's highest peaks, is renowned for its exceptional richness and endemism (Rull and Carnaval 2020), contributing significantly to the designation of the Atlantic Rainforest as a global biodiversity hotspot (Myers et al. 2000). However, the arrival of multiple climate‐tracking species into these highlands—already heavily impacted by anthropogenic disturbances that have reduced the biome to less than 10% of its original extent, largely within the Mantiqueira/Serra do Mar region (Ribeiro et al. 2009)—is likely to disrupt the delicate ecological and evolutionary dynamics that underpin their unique biotas.

Despite differences in the predicted future biodiversity distribution in the Atlantic Rainforest, both RCP 8.5 and RCP 4.5 present a concerning scenario for narrowly distributed species (considering both, RTN and FTN limits). Under the RCP 4.5 scenario, we observed significantly fewer extinctions for species with broader distributions and, consequently, less dramatic distribution shifts compared to RCP 8.5. However, the continued high extinctions of species with restricted distributions—often confined to the highlands of the Espinhaço Range and the Mantiqueira/Serra do Mar complex (Colli‐Silva et al. 2019; Rull and Carnaval 2020; Vasconcelos et al. 2020; Dantas‐Queiroz et al. 2021, 2023)—alongside the lower extinction rates and lack of climate tracking to the highlands for species with wider distributions, which are typically found at lower elevations, alter the predictions made under RCP 8.5. Therefore, under the milder RCP 4.5 projections, the highlands shift from being a target for climate‐tracking species from lower elevations to experiencing an increased species deficit, with no further species inclusion. These results underscore the concerning outlook for the Atlantic Rainforest highlands.

## 5 Conclusion

We demonstrate that increased dispersal ability correlates with broader realized and fundamental thermal niches in the Brazilian Atlantic Forest, highlighting the vulnerability of species with low dispersal capacities in the face of climate change. For these species, tracking suitable climates poleward and upward will pose significant challenges. Our findings align with the understanding that habitat specialization, narrow environmental tolerances, and limited dispersal capabilities are three major factors contributing to species vulnerability to climate change (Vié et al. 2009). To effectively understand and predict the impacts of climate change across various biogeographical regions, it is crucial to account for the dispersal abilities of local communities. This consideration will facilitate tailored management strategies for high‐ and low‐dispersal species in the tropics and beyond. We advocate that predictions regarding biodiversity shifts induced by climate change should incorporate both the environmental limits of current species ranges and, when feasible, their empirically validated physiological thresholds.

## Author Contributions

**Cleber J. N. Chaves:** conceptualization, data curation, formal analysis, funding acquisition, investigation, methodology, visualization, writing – original draft, writing – review and editing. **Ana C. Carnaval:** conceptualization, supervision, validation, writing – review and editing. **Bárbara S. S. Leal:** conceptualization, data curation, writing – review and editing. **Jessie P. Santos:** writing – review and editing. **Erison C. S. Monteiro:** conceptualization, writing – review and editing. **Clarisse Palma‐Silva:** conceptualization, investigation, resources, supervision, writing – review and editing.

## Conflicts of Interest

The authors declare no conflicts of interest.

## Supporting information


**Table S1:** Realized thermal limits of species distributions (RTN: *T*_max_ and *T*_min_) and species thermal tolerance limits (FTN: *T*_max_ and *T*_min_) for all species analyzed in this study.

**Appendix S1:** gcb70399‐sup‐0002‐AppendixS1.pdf.

## Data Availability

The data that support the findings of this study are openly available in Figshare at http://doi.org/10.6084/m9.figshare.29620895.
