# Supplementary material for: Dispersal Ability Reduces Thermal Specialization and Prevents Climate‐Driven Extinctions in a Neotropical Rainforest
Source: Glob Chang Biol. 2025 Aug 5;31(8):e70399. doi: 10.1111/gcb.70399 (PMC12323297; doi:10.1111/gcb.70399)
Supplement: Supplementary file 2 — Appendix S1: gcb70399‐sup‐0002‐AppendixS1.pdf. [file GCB-31-e70399-s001.pdf]

Dataset curation

For our datasets, we compiled 600,143 occurrence records for birds, mammals, amphibians, snakes, ants, butterflies, trees, and epiphytes from previously published sources (see Table 1 for references). When information regarding records' precision was available (i.e., Precise: the grid, transect or vegetation patch coordinates are reported. Non-precise: the coordinates of the municipality are reported, or the coordinates mismatch the written information in the reference paper; Rodrigues et al., 2019; Muylaert et al., 2017; Culot et al., 2019; Souza et al., 2019; Bovendorp et al., 2017; Nogueira et al., 2019; Vancine et al., 2018; Santos et al.. 2018; Silva et al.. 2022; Ramos et al.. 2019), we retained only the 572,598 records classified as 'precise' in the original datasets (i.e., those with coordinates not restricted to political boundaries or recorded with at least three decimal degrees of precision). From this subset, we further filtered the data to 560,965 records from 6,733 species with at least five occurrences. We included species with fewer than ten occurrences, as many endemic species in the Brazilian Atlantic Forest are naturally restricted to only a few mountaintops (e.g., Amaro et al., 2012; Firkowski et al., 2016; da Silva et al., 2018; Monge et al., 2021; Carmo et al., 2022; Vilela & Lopes, 2022) and it was a significant part of the dataset (see Fig. S2 below).

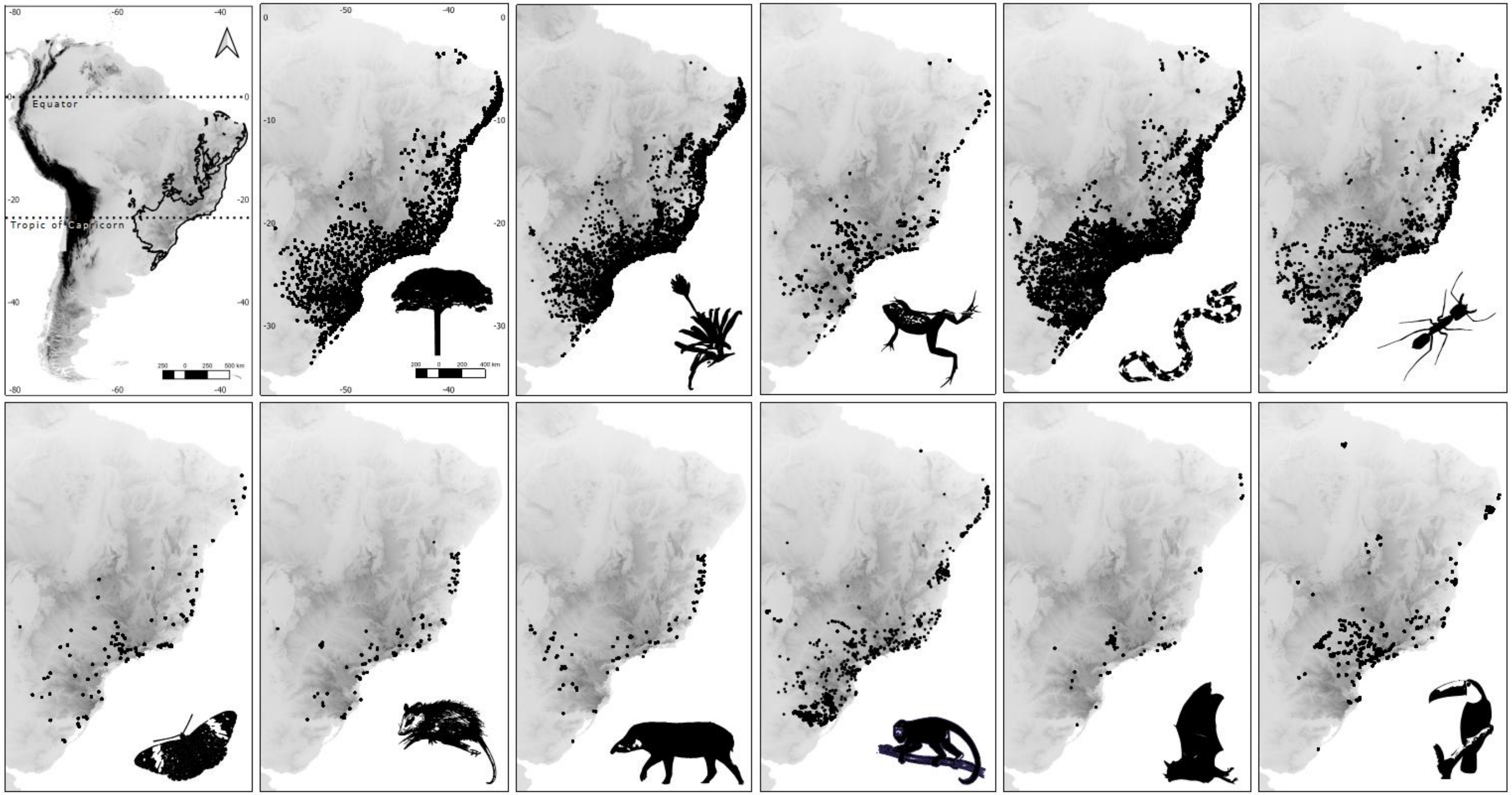

Figure S1: Geographic distribution of species occurrence records across organisms' groups. Locations of the 560,965 records for 6,732 species, encompassing trees, epiphytes, amphibians, snakes, ants, butterflies, small mammals, mid- to large-bodied mammals, primates, bats, and birds. Panels are arranged left-to-right and top-to-bottom in the order listed above.

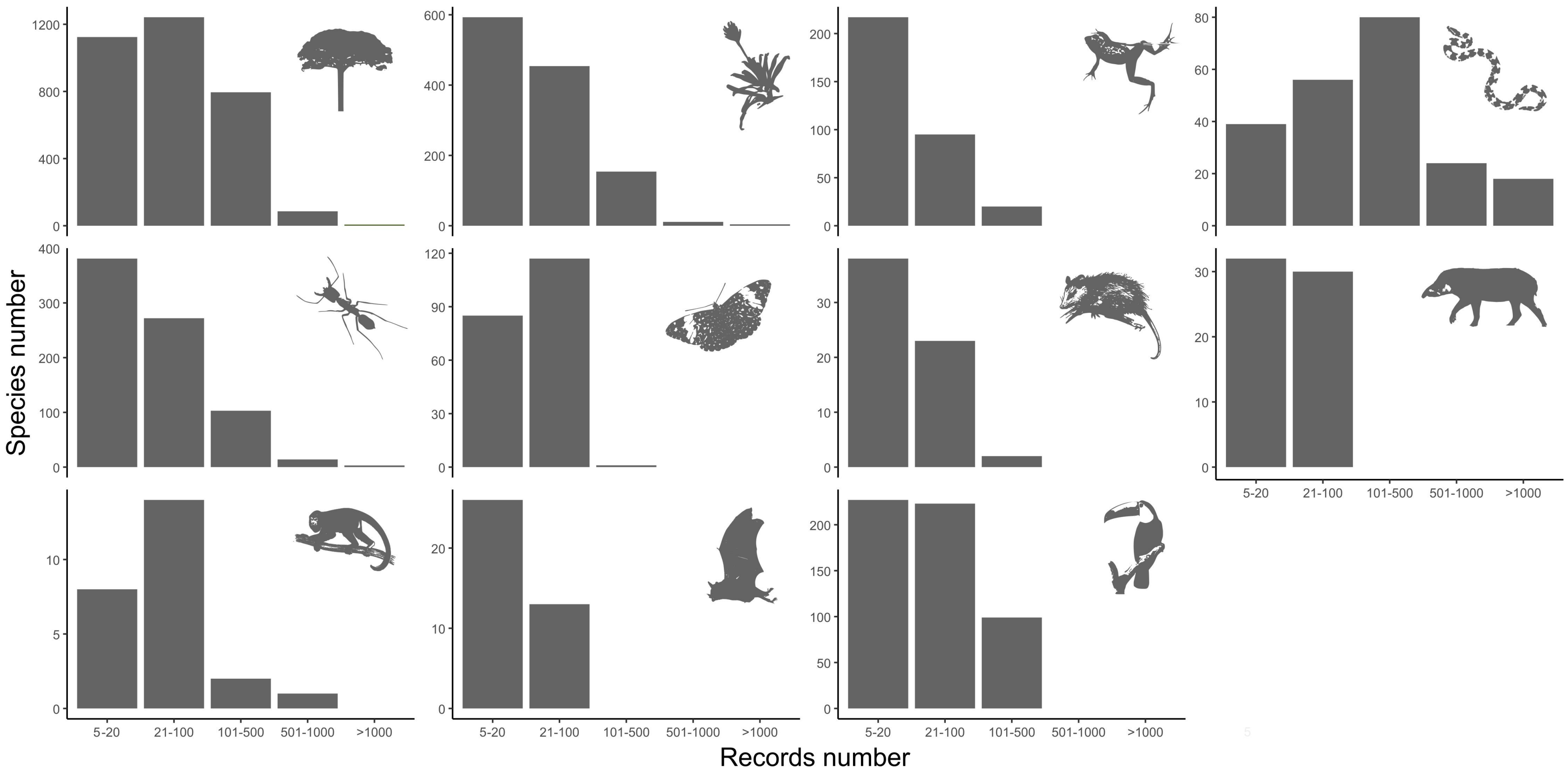

Figure S2: Frequency distribution of species records across taxonomic groups. Histograms showing the number of species (y-axis) within different record-abundance classes (x-axis) for each taxonomic group: trees, epiphytes, amphibians, snakes, ants, butterflies, small mammals, mid- to large-bodied mammals, primates, bats, and birds. Panels are arranged in left-to-right, top-to-bottom order corresponding to the sequence listed above.

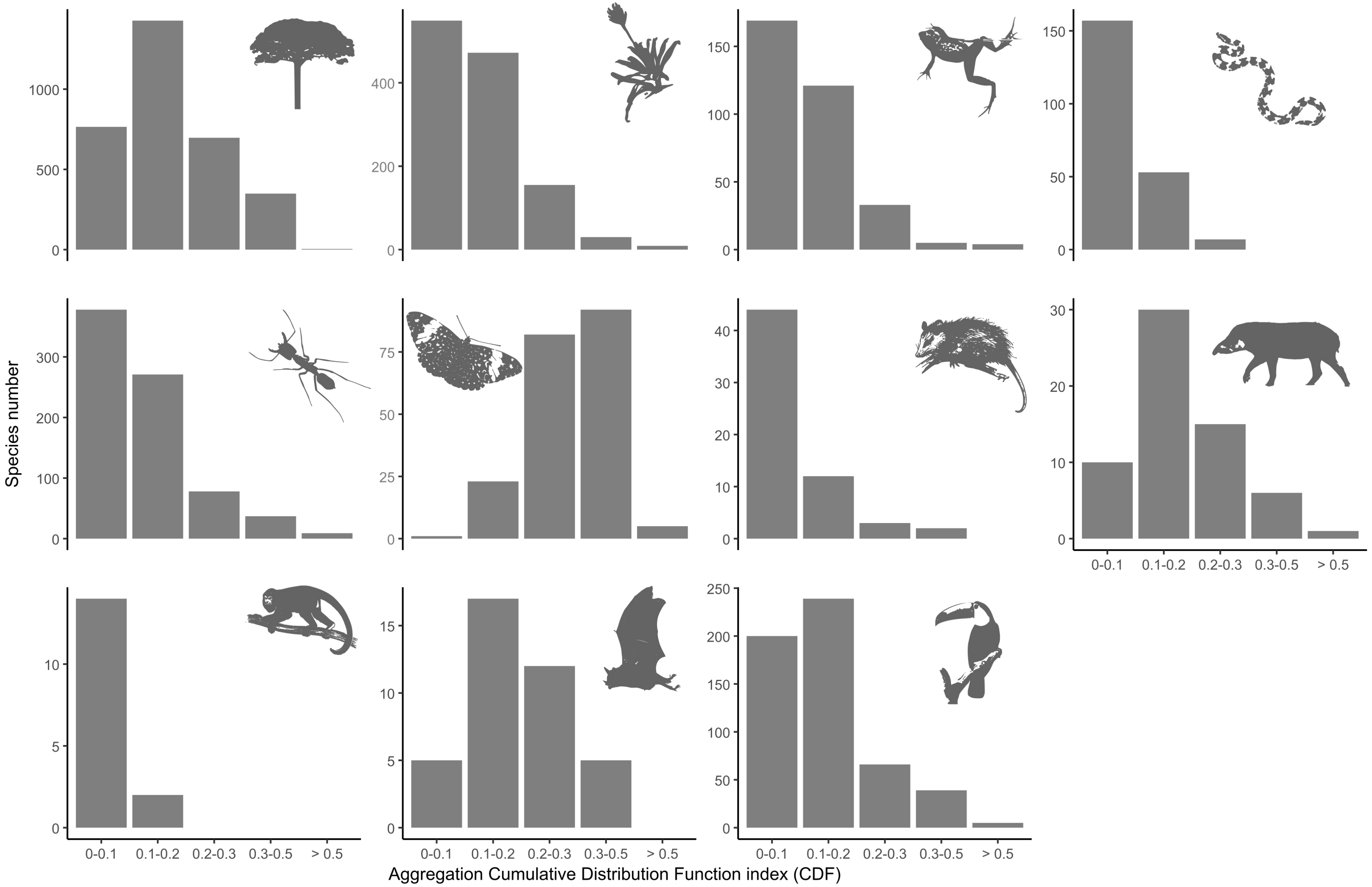

Figure S3: Frequency distribution of species records across the classes Aggregation (Cumulative Distribution Function; CDF). Lower CDF values indicate higher clustering. Histograms showing the number of species (y-axis) within different aggregation classes (x-axis) for each taxonomic group: trees, epiphytes, amphibians, snakes, ants, butterflies, small mammals, mid- to large-bodied mammals, primates, bats, and birds. Panels are arranged in left-to-right, top-to-bottom order corresponding to the sequence listed above.

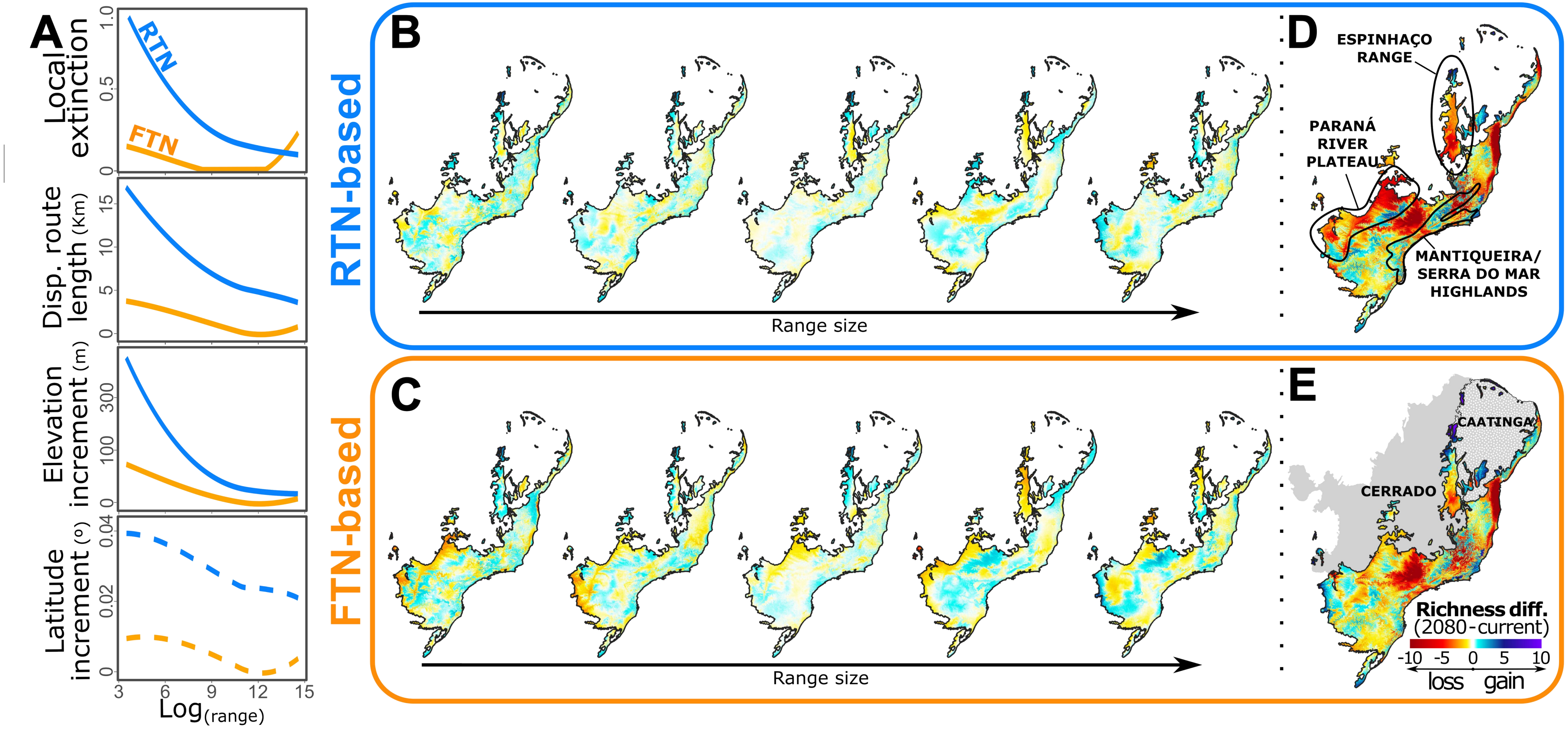

Figure S4: The effects of dispersal ability on biodiversity shifts in the Atlantic Rainforest due to climate change (RCP 4.5). Chart A presents predicted local extinction rates and climate tracking metrics—specifically dispersal route lengths, elevation and latitude increments—relative to dispersal ability (i.e., range size). The continuous and dashed curves denote significant ($p < 0.05$) and non-significant polynomial relationships for the realized (RTN; in blue) and fundamental thermal niches (FTN; in orange) of various species. Maps B-E depict the current and projected biogeographic patterns of species richness in the worst climate change scenario for the end of the 21st century (RCP 8.5; Karger et al. 2020). The current species richness maps are based on distribution data for 180 Atlantic Rainforest species with available physiological and occurrence data, while the projected maps reflect shifts arising from climate tracking indicated in Chart A. Maps in B and C differentiate expected species gain (cold colors) and loss (warm colors) by classifying species into five groups based on similar range sizes. Maps D and E present the overall patterns of species richness changes without regard to dispersal ability.
